# Treatment completion among justice-involved youth engaged in behavioral health treatment studies in the United States: A systematic review and meta-analysis

**DOI:** 10.1017/cts.2022.418

**Published:** 2022-06-13

**Authors:** Annalee Johnson-Kwochka, Eduardo F. Salgado, Casey A. Pederson, Matthew C. Aalsma, Michelle P. Salyers

**Affiliations:** 1 Department of Psychology, Indiana University-Purdue University, Indianapolis, IN, USA; 2 Adolescent Behavioral Health Research Program, Indiana University School of Medicine, Indianapolis, IN, USA; 3 Department of Pediatrics, Division of Adolescent Medicine, Indiana University School of Medicine, Indianapolis, IN, USA

**Keywords:** Adolescent, juvenile delinquency, behavioral medicine, patient participation, meta-analysis

## Abstract

Justice-involved youth (JIY) have high rates of behavioral health disorders, but few can access, much less complete, treatment in the community. Behavioral health treatment completion among JIY is poorly understood, even within treatment studies. Measurement, reporting, and rates of treatment completion vary across studies. This systematic review and meta-analysis synthesizes the literature on rates of treatment completion among JIY enrolled in research studies and identifies potential moderators. After systematically searching 6 electronic databases, data from 13 studies of 20 individual treatment groups were abstracted and coded. A meta-analysis examined individual prevalence estimates of treatment completion in research studies as well as moderator analyses. Prevalence effect sizes revealed high rates of treatment completion (pr = 82.6). However, analysis suggests a high likelihood that publication bias affected the results. Treatment groups that utilized family- or group-based treatment (pr = 87.8) were associated with higher rates of treatment completion compared to treatment groups utilizing individual treatment (pr = 61.1). Findings suggest that it is possible to achieve high rates of treatment completion for JIY, particularly within the context of family- and group-based interventions. However, these findings are limited by concerns about reporting of treatment completion and publication bias.

## Introduction

Approximately 50% of youth who have been arrested, are on probation post-adjudication, or are otherwise involved with the justice system (justice-involved youth; JIY) have a mental health disorder [1,2]. More than a third meet diagnostic criteria for a substance use disorder (SUD) [2]. Behavioral health disorders (i.e., both mental illness and SUD) are associated with an increased likelihood of recidivism and additional involvement with the justice system [3,4]; further justice system involvement, in turn, is associated with higher rates of behavioral health disorders [2]. While treatment completion has been associated with positive health and life outcomes (i.e., employment, housing) in substance use treatment [5] and with reduced recidivism among JIY youth specifically [6], JIY often do not complete available treatment for behavioral health disorders [7,8], even in the context of well-resourced treatment studies [9–11]. It is critical to understand factors involved in treatment completion among JIY.

Data suggest that JIY and their families experience challenges in completing treatment for behavioral health disorders [12]. For example, 2017 data from the Substance Abuse and Mental Health Services Administration indicate that only 45% of youth aged 12–20 years who were referred to publicly funded substance abuse treatment by a criminal justice organization successfully completed treatment [13] – most dropped out of treatment early or were discharged by the service provider due to lack of compliance with treatment. A recent cross-sectional analysis of administrative data from the Florida Department of Juvenile Justice suggests that, although 32% of the sample met criteria for SUD treatment, only 11.5% completed a SUD treatment program [14].

While administrative data may show the extent of the problem, they provide little understanding of how to address the problem. However, treatment studies for JIY may provide more insight. These studies often provide high-quality, evidence-based care, or are testing new interventions. In these contexts, researchers often make considerable efforts to help participants and their families maintain participation [15], and may therefore represent the best-case scenario for measuring treatment completion among JIY. A better understanding of the factors that contribute to successful completion in these contexts may help inform future research and practice.

### Influences on treatment completion among JIY

Existing research suggests a number of possible moderators of behavioral health treatment completion among JIY. A recent systematic review examined empirical evidence on the effects of three types of interventions designed to improve engagement in behavioral health treatment among adolescents (not exclusively focused on JIY): systems-level (e.g., offering treatment services in-home), family-level (e.g., informing family members about treatment topics), or individual-level (e.g., utilizing contingency management interventions) [16]. Findings suggested that any type of intervention designed to increase behavioral health treatment engagement has positive influences on attendance at varying stages of treatment. Type of treatment (i.e., group, individual, family) may also have an influence on the extent to which youth are able to engage in and complete treatment; existing research suggests that family-based treatments are associated with greater engagement in substance use treatment among adolescents [17]. JIY who are members of ethnic and/or racial minority groups may be less likely to have access to or utilize mental health treatment [8]; research also finds that JIY of color have lower rates of treatment completion [14]. Some have argued that these disparities might reflect a difference in needs – for example, Black JIY are at an increased risk of experiencing poly-victimization, defined as having experienced *many* different types of traumatic victimization in their lifetime including assault, family/community violence, physical or sexual abuse, and trauma from racially driven encounters [18,19]. JIY of color are also more likely to experience a wide array of comorbid mental disorders [20], further demonstrating their need for high quality treatment. Researchers have argued that current treatment options may not be properly poised to address the complex stressors that JIY of color experience [19,21], thus discouraging engagement with treatment. Despite this, many youth of color do not have access to quality treatment options due to the fact that these youth tend to live in poorer communities with fewer resources, a systematic barrier [20]. Other barriers unique to engaging JIY youth of color include cultural mistrust of healthcare services rooted in historical oppression [22], greater logistical challenges (e.g., transportation, insurance issues) [20], and difficulties engaging family members in treatment (e.g., language barriers, competing demands) [20].

Low rates of treatment completion among JIY may be surprising, since JIY often enter treatment because of legal mandates (e.g., as a condition of probation). However, the effect of juvenile drug courts – one of the most common examples of mandated treatment – has been highly variable, especially in comparison to more commonly successful adult drug courts [23]. Current evidence suggests that most juvenile drug courts minimally engage parents and youth; a meta-analytic review found that slightly more than half of all youth who initially enroll in a juvenile drug court program end up graduating and that youth who enroll but do not graduate (i.e., are terminated unsuccessfully) do not appear to benefit from participating in the program based on later measures of substance use and recidivism [24]. Given these findings, researchers conclude that it is necessary to implement additional efforts to engage youth in treatment, beyond or instead of court mandates [24]. Especially given the increase in diversion of youth away from the justice system, it is critically important to understand how to constructively engage youth without the force of court mandates [25]; the first step in this process is understanding complexities in current rates of treatment completion among JIY and identifying potential moderators.

### Purpose of Study

Longitudinal studies of behavioral health treatment among JIY report inconsistent findings, with wide ranges in rates of treatment completion [26–28] even in the context of additional resources available to researchers to help engage participants. The current study aims to conduct a meta-analysis to quantify treatment completion among JIY enrolled in behavioral health treatment studies. In addition, the current study aims to determine whether demographic variables (i.e., gender, race/ethnicity) and methodological variables (i.e., intervention focus, type of treatment, the presence or absence of interventions to increase treatment engagement) moderate the prevalence of treatment completion among JIY.

## Methods

Procedures and results are reported in accordance with the Preferred Reporting Items for Systematic Reviews and Meta Analyses (PRISMA) Statement [29,30], which is available in Table 1.

**Table 1.** PRISMA reporting checklist for systematic reviews and meta-analysis

Section/topic	#	Checklist item	Reported on page #
Title			
Title	1	Identify the report as a systematic review, meta-analysis, or both.	1
Abstract			
Structured summary	2	Provide a structured summary including, as applicable: background; objectives; data sources; study eligibility criteria, participants, and interventions; study appraisal and synthesis methods; results; limitations; conclusions and implications of key findings; systematic review registration number.	2
Introduction			
Rationale	3	Describe the rationale for the review in the context of what is already known.	3 - 6
Objectives	4	Provide an explicit statement of questions being addressed with reference to participants, interventions, comparisons, outcomes, and study design (PICOS).	5 - 6
Methods			
Protocol and registration	5	Indicate if a review protocol exists, if and where it can be accessed (e.g., Web address), and, if available, provide registration information including registration number.	NA
Eligibility criteria	6	Specify study characteristics (e.g., PICOS, length of follow-up) and report characteristics (e.g., years considered, language, publication status) used as criteria for eligibility, giving rationale.	6
Information sources	7	Describe all information sources (e.g., databases with dates of coverage, contact with study authors to identify additional studies) in the search and date last searched.	7
Search	8	Present full electronic search strategy for at least one database, including any limits used, such that it could be repeated.	7, Table 2
Study selection	9	State the process for selecting studies (i.e., screening, eligibility, included in systematic review, and, if applicable, included in the meta-analysis).	6–7
Data collection process	10	Describe method of data extraction from reports (e.g., piloted forms, independently, in duplicate) and any processes for obtaining and confirming data from investigators.	7
Data items	11	List and define all variables for which data were sought (e.g., PICOS, funding sources) and any assumptions and simplifications made.	7 - 9
Risk of bias in individual studies	12	Describe methods used for assessing risk of bias of individual studies (including specification of whether this was done at the study or outcome level), and how this information is to be used in any data synthesis.	9
Summary measures	13	State the principal summary measures (e.g., risk ratio, difference in means).	9
Synthesis of results	14	Describe the methods of handling data and combining results of studies, if done, including measures of consistency (e.g., I [2]) for each meta-analysis.	9–11
Risk of bias across studies	15	Specify any assessment of risk of bias that may affect the cumulative evidence (e.g., publication bias, selective reporting within studies).	11
Additional analyses	16	Describe methods of additional analyses (e.g., sensitivity or subgroup analyses, meta-regression), if done, indicating which were pre-specified.	10 - 11
Results			
Study selection	17	Give numbers of studies screened, assessed for eligibility, and included in the review, with reasons for exclusions at each stage, ideally with a flow diagram.	7, 11, Fig. 1
Study characteristics	18	For each study, present characteristics for which data were extracted (e.g., study size, PICOS, follow-up period) and provide the citations.	11–13, Table 3 and 4
Risk of bias within studies	19	Present data on risk of bias of each study and, if available, any outcome-level assessment (see item 12).	13
Results of individual studies	20	For all outcomes considered (benefits or harms), present, for each study: (a) simple summary data for each intervention group and (b) effect estimates and confidence intervals, ideally with a forest plot.	13, Fig. 2
Synthesis of results	21	Present results of each meta-analysis done, including confidence intervals and measures of consistency.	13, Table 5
Risk of bias across studies	22	Present results of any assessment of risk of bias across studies (see Item 15).	14
Additional analysis	23	Give results of additional analyses, if done (e.g., sensitivity or subgroup analyses, meta-regression [see Item 16]).	13
Discussion			
Summary of evidence	24	Summarize the main findings including the strength of evidence for each main outcome; consider their relevance to key groups (e.g., healthcare providers, users, and policy makers).	14- 15, 18
Limitations	25	Discuss limitations at study and outcome level (e.g., risk of bias), and at review-level (e.g., incomplete retrieval of identified research, reporting bias).	16
Conclusions	26	Provide a general interpretation of the results in the context of other evidence and implications for future research.	15–18
Funding			
Funding	27	Describe sources of funding for the systematic review and other support (e.g., supply of data); role of funders for the systematic review.	NA

### Inclusion and Exclusion Criteria

Studies were included if: 1) they were available in English, 2) the sample included youth who are involved in the juvenile justice system and who resided in the community at the time of the study (e.g., youth on probation, youth who have been arrested and then diverted from the justice system through a diversion program), 3) the study included an assessment to determine eligibility for treatment and the provision of a behavioral health treatment, and 4) the authors reported criteria for treatment completion. Studies in which all JIY were eligible (i.e., the primary goal of services was to prevent recidivism) were excluded; however, studies targeting behavioral problems (i.e., youth adjudicated through drug court, youth who sexually offend) indicative of a specific behavioral health disorder being treated were included. Book chapters and dissertations were included in literature searches. Studies were excluded if they were cross-sectional, were primarily studies of behavioral health service utilization (i.e., assessing whether youth access treatment not provided as part of the study), or represented evaluations of treatment services in which only participants who completed treatment were included in analyses. Studies that combined youth who were and were not JIY or included both adult and juvenile participants were excluded unless information for only the JIY participants could be obtained.

### Literature Search

**Fig. 1.**
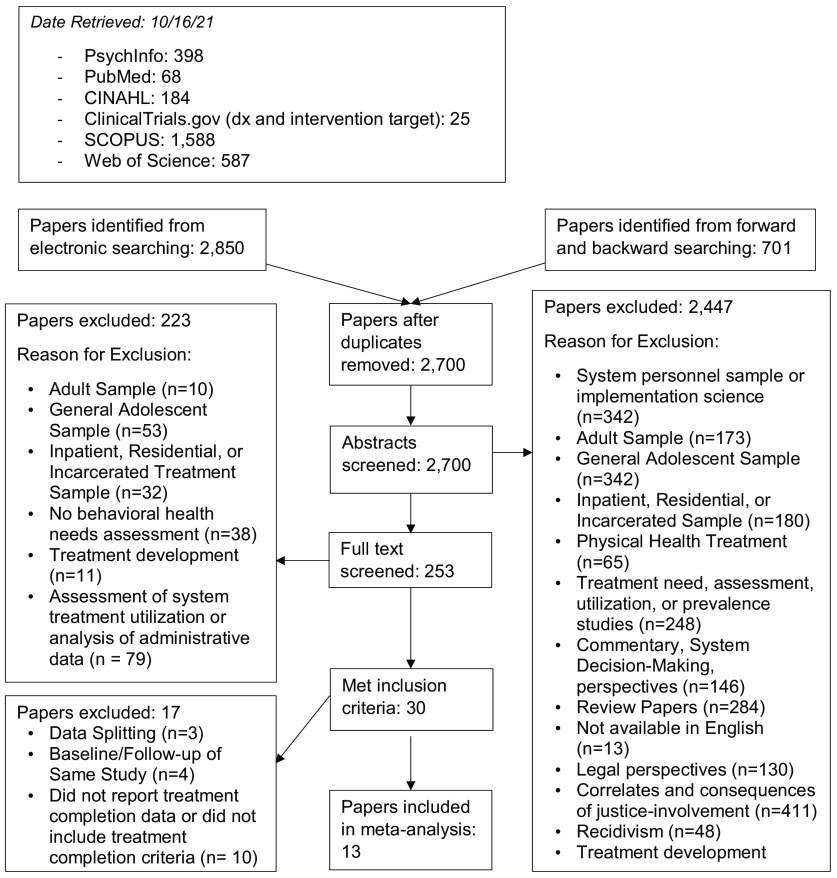
PRISMA flow chart.

We identified studies meeting criteria for inclusion using four concurrent methods. First, we identified 2,850 studies through database literature searches (see Table 2 for databases and search terms). Second, because the terminology to identify JIY is quite heterogeneous (e.g., justice-involved, juvenile offender, probationers, delinquent, justice-referred), we identified 701 articles through extensive forward- and backward-literature searching of reviews on behavioral health treatments for adolescents and reviews of interventions for JIY [31–47]; this is an established method of conducting literature searches in areas where the terminology is not standardized [48]. Third, subject matter experts were contacted (i.e., authors of published articles on studies funded by JJTrials) to inquire about data from unpublished studies, but no additional studies were obtained from these contacts. Finally, we created email alerts in Google Scholar for the above search; no newly eligible studies were identified prior to manuscript submission. After duplicates were removed, we reviewed the title and abstract of 2,700 articles with the full text of 253 articles being reviewed (see Fig. 1 for PRISMA flow chart and article exclusions). Thirteen studies were coded for inclusion in the data analysis.

**Table 2.** Database search terms

Database	Search Terms
PSYCHInfo	((MM “Substance Use Treatment” OR MM “Alcohol Treatment” OR MM “Mental Health Services” OR MM “Community Mental Health Services”) AND (MM “Juvenile Delinquency” OR MM “Juvenile Justice”) using PSYCHInfo subject headings thesaurus
CINAHL	((MM “Substance Use Rehabilitation Programs+” OR MM “Mental Health Services+”) AND (MM “Juvenile Offenders+” OR MM “Juvenile Delinquency”)) using CINAHL subject headings
PubMed	((“juvenile delinquency” [MeSH Terms]) AND ((“substance-related disorders“[MeSH Terms]) OR (“mental processes/psychology“[MeSH Terms])) AND (“treatment outcome“[MeSH Terms])) using MeSH terms
ClinicalTrials.gov	(“juvenile justice” OR “juvenile delinquent”) AND (“treatment” OR “intervention” OR “therapy”) | (“Mental health disorder” OR “substance use disorder”)
SCOPUS	((“juvenile justice” OR “juvenile delinquent”) AND (“mental health treatment” OR “substance use treatment” OR “behavioral health treatment” OR “mental health intervention” OR “substance use intervention” OR “behavioral health intervention”))
Web of Science	(ALL = (adolescent OR youth OR juvenile) AND ALL = (“juvenile justice” OR “juvenile delinquent” OR probation OR diversion OR diverted) AND ALL = (“mental health treatment” OR “substance use treatment” OR “behavioral health treatment” OR “mental health intervention” OR “substance use intervention” OR “behavioral health intervention” OR therapy)) AND LANGUAGE: (English)

### Coding

We coded studies according to a fixed coding protocol, following guidelines from Lipsey, Wilson [49], and Card [50]; variables included study characteristics, primary outcomes of interest, moderators, and study quality. Each study was coded independently by the first, second, and third authors; after coding, the authors met to identify and resolve discrepancies in coding.

### Prevalence Estimates

We coded the number of JIY who began treatment and the number who completed treatment according to the study’s stated treatment completion criteria. This prevalence rate was coded as *treatment completion*. If studies reported sufficient data on multiple treatment conditions, separate effect sizes were estimated for each treatment condition. For RCTs where “treatment completion” criteria were not specified for the control condition (usually “services as usual”), the treatment completion prevalence rate was only coded for the experimental condition(s).

### Moderators

#### Racial and ethnic minority participants

The prevalence of racial and ethnic minority participants was coded as a percentage for each study or treatment group and included in analysis of moderators.

#### Intervention Focus

We assessed intervention focus as a moderator; this variable was coded as 1 = substance use disorders and 2 = other behavioral health for analyses.

#### Type of Treatment

Type of treatment was coded as 1 = individual, 2 = group, and 3 = family for analyses. When treatments included multiple components (e.g., individual sessions with the youth as well as sessions with youth and parent(s), individual therapy with group skills training classes), it was coded according to the study’s identified type of treatment (e.g., a family-based treatment) and the highest level of clinical intensity in the treatment. Thus, if a treatment included both individual and family components and was described as family-based treatment, it was coded as 3 = family. If a treatment included individual therapy sessions and less-frequent group skill-building classes, it was coded as 1 = individual.

#### Interventions to Increase Treatment Engagement

The presence or absence of reported interventions to increase treatment engagement was coded as a moderator (1 = present, 2 = not present). Interventions to increase engagement commonly included contingency management programs, engaging family in the treatment planning process, providing treatment in the youth’s home or other convenient locations, providing transportation to treatment, or a comprehensive assessment of barriers to engagement and subsequent problem solving that focuses on the whole ecology of youths and families, as is standard in multisystemic therapy (MST). Basic phone or text-message reminders of treatment appointments, as are commonly provided in behavioral healthcare, were not coded as interventions to increase engagement.

#### Treatment Mandates

Whether or not youth were mandated by a court to participate in outpatient treatment was coded as the percentage of youth in the study who were required to participate in outpatient treatment.

#### Primary Study Quality

Standardized measures for assessing primary study quality in systematic reviews and meta-analyses typically focus on specific study designs (e.g., quasi-experimental studies, noncontrolled longitudinal studies) and are geared toward assessing risk of bias in the study’s main outcome variable. Because we were primarily interested in judging the quality of measures of attendance and attrition (rather than bias in outcome) across studies with varied designs, we developed a checklist after reviewing existing measures of study quality [51–53] as well as review articles on the measurement of treatment engagement [16,17,54,55]. The checklist was composed of 5 yes/no questions for all primary studies and 2 additional questions for studies with randomized designs; the checklist is available as a Supplementary file.

### Data Analysis

#### Effect Size Calculations

All analyses were performed in R (Version 4.1.3) with packages Metafor and Meta [56–58]. To assess proportional data, an analysis of binary outcomes was pooled in the form of proportions with a generalized linear mixed model (GLMM) using the logit link function with Clopper-Pearson intervals to stabilize the variance [59,60]. Simulation studies indicate that the GLMM model provides the most accurate estimate in proportional meta-analysis because GLMM models do not require data transformations within studies [61,62], fully account for uncertainties, and produce confidence intervals with satisfactory coverage probabilities [63]. We implemented all parameters via the maximum likelihood approach. Random effects models were selected to calculate effect sizes because they represent a more conservative estimate of mean prevalence and to account for heterogeneity between studies.

We examined forest plots to identify potential outliers, i.e., studies whose individual 95% CI did not overlap with the 95% CI for the mean effect [64,65]. Potential outliers were removed from calculation of effect size if overall prevalence rates were affected [65]. Heterogeneity of the studies was assessed using Cochran’s Q-Test, which tests for the presence of heterogeneity across studies [66], and Higgins I^2^, which describes the percent of variation in prevalence across studies due to heterogeneity rather than chance [67]. Low heterogeneity was defined as Q scores < critical chi square values and I^2^ < 25%; moderate if Q > critical chi square values and I^2^ around 50%; and high if Q > critical chi square values and I^2^ > 50%. Statistical significance was defined as *P* < 0.05 [67].

### Moderator Analyses

If significant heterogeneity of individual prevalence estimates was found via the two criteria, mixed-effect meta regression was used to attempt to explain the between-study heterogeneity based on study-level fixed-effect covariates (i.e., subgroups defined by categorical covariates or continuous covariates). Specifically, candidate variables were tested to identify significant moderators (i.e., candidate variables that account for a significant proportion of variability in individual prevalence across studies) in nonlinear mixed-effects models, such that random-effect terms were used to combine studies within each subgroup, and fixed-effect terms were used to combine subgroups and yield the overall effect [68]. Study-to-study variance (*T* ^2^) was not assumed to be the same for all subgroups; the value was computed within subgroups [69]. The *Q*_between_ statistic (analogous to analysis of variance) tested categorical variables to report between-study variance explained by moderators. We calculated mean effect sizes within all variable levels [70]. Variables were considered moderators if the mixed(random)-effects model indicated statistical significance (*P* < 0.05) on the *Q*_between_ statistic [71]. Interactions among moderator variables were not tested due to insufficient power.

### Publication Bias

Publication bias was assessed with funnel plot symmetry both visually and statistically by using Egger’s linear regression method to assess any relationship between sample size and prevalence [72]. If significant funnel plot asymmetry was present, the trim and fill method was used to determine the number of missing studies that would be needed to correct the asymmetry [73]. An additional quantitative assessment of bias used the Begg’s rank method [74] to identify relationships between effect sizes and sample sizes. Low publication bias was deemed present if funnel plots were visually symmetrical and were not statistically significant. Finally, given the gaps in the ability to assess publication bias in proportional meta-analyses using established statistical methods, we offer qualitative assessments of the role of publication bias in these analyses.

## Results

### Description of Included Studies

Altogether, 13 studies [9–11,26,28,75–82] representing 20 treatments (e.g., services as usual, Multisystemic Therapy) met inclusion criteria (see Table 3). Three of the 13 studies included services as usual treatment conditions in which they did not specify treatment completion criteria (see Table 4 for details); adolescents in these groups were not included in the tables or in the descriptions below. All studies were peer-reviewed published articles and were located in the United States. Complete descriptions of all 13 studies are included in Tables 3 and 4.

**Table 3.** Effect size variables and demographics

	Effect Size Variables					Demographics		
	Eligible N	Initiation N	Treatment Group	Treatment Group N	Completion N	Age (SD)	% Minority	% Female
Borduin et al., 2009	51	48	MST (T)	24	24	14 (1.9)	29.2	4.2
			SAU (C)	24	22			
Burrow-Sanchez et al., 2015	71	70	Standard CBT (T1)	36	24	15.3 (1.3)	100.0	11.1
			Accommodated CBT (T2)	34	27	15.1 (1.2)	100.0	8.8
Dakof et al., 2015	119	112	MDFT (T1)	55	53	16.0 (1.1)	100.0	10.9
			AGT (T2)	57	49	16.1 (0.9)	100.0	10.5
Henderson et al., 2016	145	126	ACRA (T)	63	40	15.1 (1.1)	25.2	20.6
			SAU^*^	63	not reported	15.3 (1.0)	22.0	26.9
Henggeler et al., 1999	141	118	MST (T)	58	57	15.7 (1.0)	53.0	21.1
			SAU (C)	60	3			
Henggeler et al., 2015	115	104	CM (T)	73	63	15.4 (0.9)	43.5	17.6
			SAU (C)	42	41			
Kaminer et al., 2019	142	113	MET-CBT (T)	NA	102	16.1 (1.0)	62.3	12.1
Letourneau et al., 2009	178	131	MST (T)	68	62	14.6 (1.7)	85.0	2.4
			SAU (C)	66	58			
Schaeffer et al., 2014	104	97	Community Restitution (T)	50	33	15.8 (0.9)	82.0	17.0
			SAU^*^	47	not reported	15.9 (0.9)	85.0	18.0
Sharkey et al., 2010	not reported	150	Neighborhood enrichment (T)	NA	150	16.02 (1.2)	80.9	38.0
Silovsky et al., 2019	418	320	CBT (T)	NA	188	12.8 (1.6)	not reported	9.1
Tolou-Shams et al., 2017	233	60	Family Affect Management(T1)	30	25	15.6 (1.3)	50.7	30.0
			Health Promotion (T2)	30	22			
Walker et al., 2019	136	101	Girls Active Learning	66	57	15.2 (1.7)	41.0	100.0
			SAU^*^	35	not reported			

Note: In some cases, authors reported demographics for all participants (rather than by treatment group) after finding that there were no significant demographic differences between treatment groups.

Abbreviations: Multisystemic Therapy (MST), Services as Usual (SAU), Cognitive Behavioral Therapy (CBT), Multidimensional Family Therapy (MDFT), Motivational Enhancement Therapy (MET), Adolescent Group Treatment (AGT), Adolescent Community Reinforcement Approach (ACRA), Contingency Management (CM).

* SAU treatment groups that did not define or report treatment completion data were not included in analyses.

**Table 4.** Methodological variables

	Methodological Variables							
	Study Design	Intervention Focus	Justice-Involvement Type	Treatment Mandates	Type of Treatment	Engagement Interventions	Treatment Length	Completion Criteria
Borduin et al., 2009	RCT	Problematic Sexual Behavior	arrested; could be pre- or post- adjudication	Court-ordered outpatient sexual offender counseling	MST; family (T)	Services provided in convenient location; therapists available 24/7	Averaged 30 weeks	Collaboratively identified by MST therapist and patient
					SAU; individual (C)	None reported	Averaged 30 weeks	Collaboratively identified by MST therapist & patient
Burrow-Sanchez et al., 2015	RCT	Any SUD	heterogeneous; all participants referred by juvenile probation department	74% court-ordered to treatment	Standard-CBT; group (T1)	None reported	12 weeks	Attended 9 of 12 treatment sessions
					accommodated CBT; family (T2)	Treatment manual modified to increase relevancy for Latino adolescents and include family treatment; promoted regular phone/mail contact between therapist and patients/families	12 weeks	Attended 9 of 12 treatment sessions
Dakof et al., 2015	RCT	Any SUD	All participants enrolled in drug court	Court-ordered to treatment	MDFT; family (T1)	Financial assistance with transportation; services provided in convenient location; included family in treatment planning	Averaged 20 weeks	Graduation from drug court
					Adolescent Group Treatment; group (T2)	Financial assistance with transportation; therapists reached out to DC and family if youth failed to attend	Averaged 20 weeks	Graduation from drug court
Henderson et al., 2016	RCT	Any SUD	On probation	Not reported	Adolescent-Community Reinforcement Approach; individual (T)	Financial assistance with transportation; services provided in convenient location	Averaged 36 weeks	Attended 3 or more sessions
					SAU; individual^*^	None reported	Averaged 36 weeks	Not specified
Henggeler et al., 1999	RCT	Any SUD	Formal or informal probation	Not reported	MST; family (T)	Services provided in convenient location; therapists available 24/7	Averaged 18 weeks	Collaboratively identified by MST therapist & patient
					SAU; individual (C)	None reported	Not Described	Collaboratively identified by MST therapist & patient
Henggeler et al., 2015	RCT	Problematic drug use	All participants enrolled in drug court	Court-ordered to treatment	Contingency management – family engagement; family (T)	Contingency management intervention; included family in treatment planning	Averaged 20 weeks	Graduation from drug court
					SAU; group (C)	None reported	Averaged 12 weeks	Graduation from drug court
Kaminer et al., 2019	Non-controlled	Cannabis use disorder	All participants referred by juvenile probation department	Not reported	MET-CBT; individual (T)	None reported	7 weeks + additional 10 weeks for non-responders	Attending at least the first and last session of each phase
Letourneau et al., 2009	RCT	Problematic sexual behavior	On probation or in a diversion program	Court-ordered outpatient sexual offender counseling	MST; family (T)	Services provided in convenient location; therapists available 24/7	Averaged 28.4 weeks	Collaboratively identified by MST therapist & patient
					SAU; group (C)	None reported	Averaged 50 weeks	Collaboratively identified by MST therapist & patient
Schaeffer et al., 2014	RCT	Any SUD	All participants referred by juvenile probation department	Not reported	Community restitution apprentice-focused training; individual (T)	Financial assistance with transportation; individualized program attendance schedules coordinated with schools	6 months	Attendance for a minimum of 100 hrs of instruction & proficiency in all core skill areas
					SAU; individual^*^	None reported	Not described	Not specified
Sharkey et al., 2010	Non-controlled	Any SUD	Identified for services through juvenile probation or truancy	Not reported	Neighborhood enrichment with vision involving services, treatment, and services; family	Services centrally located for easier transportation; culturally modified treatment by recruiting providers from community	Not described	Completion of curriculum
Silovsky et al., 2019	Non-controlled	Problematic sexual behavior	Juvenile probation actively involved in all treatment teams; participants on probation or diverted	42% court-ordered to treatment	CBT with concurrent groups for youth and caregivers; family (T)	None reported	Not described	Completion of curriculum
Tolou-Shams et al., 2017	RCT	Problematic drug use	All participants enrolled in drug court	Not reported; notes that court was unaware of youth participation in study	Family-based Affect Management Intervention; family (T1)	None reported	7 weeks	Completed core intervention (4 weeks)
					Adolescent-only Health Promotion Intervention; individual (T2)	None reported	7 weeks	Completed core intervention (4 weeks)
Walker et al., 2019	Quasi-experimental	Problematic drug use	On probation	Not reported	Girls Only Active Learning; group (T)	Weekly text messages to parents about attendance and the weekly program topic	10 weeks	Completed if youth did not miss more than 3 sessions
					SAU; individual^*^	None reported	Not described	Not specified

Abbreviations: Multisystemic Therapy (MST), Services as Usual (SAU), Cognitive Behavioral Therapy (CBT), Multidimensional Family Therapy (MDFT), Motivational Enhancement Therapy (MET).

* In some studies, Services as Usual (SAU) conditions did not report treatment completion criteria or data. These conditions were not included in analyses.

The 20 eligible treatment conditions included a total sample size of 1,269 adolescents, with a mean sample size of 74.7 (SD = 70.7, range = 24–320). Samples averaged 15.2 years of age, were predominantly male (80.2%), and were predominantly from minority ethnic or racial populations (68.1%). Studies were primarily focused on substance use disorders (*n* = 10, 76.9%). Individual treatment conditions utilized different types of treatment, categorized as family (*n* = 9, 52.9%), individual (*n* = 4, 23.5%), or group (*n* = 4, 23.5%). When treatment length and completion criteria were reported, treatments were designed to last an average of 18.4 weeks (SD = 8.6).

Studies employed a wide range of strategies to increase treatment completion; of 20 individual treatment groups (including services as usual and experimental treatment conditions, which often used different treatment engagement strategies between groups), 11 (65%) reported employing interventions to increase treatment engagement; see Table 4. Six treatment groups provided services in locations convenient to the youth or family (e.g., home, school, community spaces). Four treatment groups offered financial assistance with transportation to treatment, three made on-call therapists available to families at all times, and 4 included family in-treatment planning (e.g., contacting family each week to describe the group session topic, encouraging regular contact between therapists and families). Two treatment groups modified services to be culturally adapted, e.g., by recruiting providers from the local community. Finally, only one treatment group utilized a contingency management intervention to increase youth attendance in treatment. Nine treatment groups did not specify any treatment engagement strategies. See Table 4 for descriptions of engagement strategies used in each study.

Supervision or JIY court involvement varied widely both between and within studies; youth were on probation, arrested and entering treatment pre-adjudication, in formal diversion programs, or enrolled in drug court. In three studies (23.1%), participants were recruited entirely from juvenile drug courts. Whether or not youth had been mandated to participate in treatment also varied widely both within and between studies; 7 studies (53.8%) did not report information on treatment mandates; see Table 4.

### Treatment Completion

**Fig. 2.**
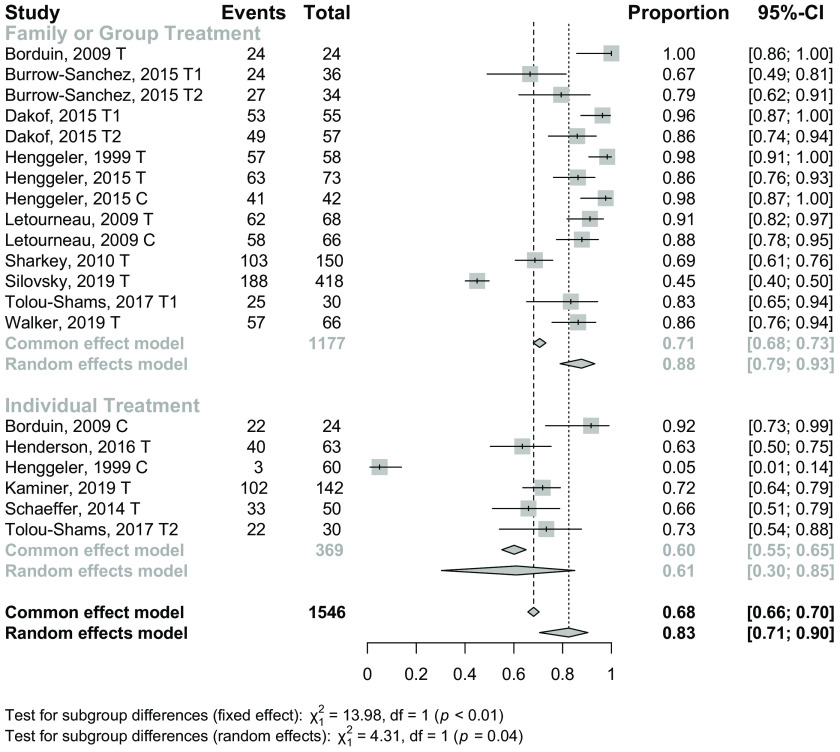
Treatment completion by treatment type. Note: Treatment groups are marked as T (treatment group), C (control group), or T1 and T2 for multiple treatments in comparative effectiveness studies.

A total of 13 studies yielded 20 individual prevalence estimates; see Fig. 2 for the forest plot of effect sizes and Table 5 for associated model statistics. Although there were two significant outliers from the mean prevalence estimate [9,77], neither of these individual estimates had a significant effect on the overall estimate when removed from analyses, so individual estimates from all studies were retained. The main effect size for treatment completion was pr = 82.6 (CI = 70.6, 90.3), indicating that approximately 82.6% of JIY completed treatment, as defined by each study’s individual treatment completion criteria. The heterogeneity in individual prevalence estimates across studies was high (Q = 221.3, I^2^ = 91.4), meeting criteria for moderation analyses.

**Table 5.** Effect sizes and associated statistics

Full model results for meta-analysis								
	Number of effects	Effect Size	95% CI	Range	*Q* Statistic	I^2^ Index	Begg’s rank correlation	Egger’s weighted regression
Treatment Completion	20	82.6	70.6, 90.3	5.0–98.0	221.3	91.4	0.08	< 0.01
Mixed effects models for analysis of categorical moderators								
	*k*	Effect Size	95 % CI	Range	*Q*_*between*_ Statistic	*df*	*P*	
*Intervention Focus*								
SUD	15	80.0	66.7, 96.9	1.6–99.7	0.63	1	0.39	
Other	5	88.7	66.7, 96.9	40.3–99.9				
*Treatment Type*								
Family or Group	14	87.8	78.9, 93.2	38.0–99.9	4.31	1	0.04	
Individual	6	61.1	30.4, 84.9	1.6–97.9				
*Interventions to Increase Treatment Engagement*								
Absent	9	75.9	49.7, 90.1	1.6–99.7	0.9	1	0.33	
Present	11	86.0	75.4, 92.5	50.0–99.9				
Meta-regression models for analysis of continuous moderators								
	Coefficient	Standard Error	95% CI				*P*	
% Minority	0.17	0.13	–0.09, 0.42				0.21	

### Moderator Analyses

Subgroup analyses revealed that treatment completion was higher in studies that provided family- or group-based treatment (pr = 87.8, CI = 78.9, 93.2, k = 14) compared to studies providing individual treatment (pr = 61.1, CI = 30.4, 84.9, k = 6); see Fig. 2 for a forest plot of effect sizes by type of treatment and Table 5 for full results from moderator analyses. No other significant moderators were identified.

### Primary Study Quality

Studies included a range of designs: 9 studies (52.9%) were RCTs (some of these employed a cluster-randomized design), one (7.7%) was a quasi-experimental trial with a control group, and three (23.1%) were noncontrolled longitudinal trials of treatment effectiveness (see Supplemental Table). All but one study conducted intent-to-treat analyses, in which all participants who entered treatment were included in outcome analyses. Only three studies (23.1%) provided a detailed description of youth who did not complete treatment, including reasons for attrition and demographic characteristics of those who did not complete treatment.

### Publication bias

**Fig. 3.**
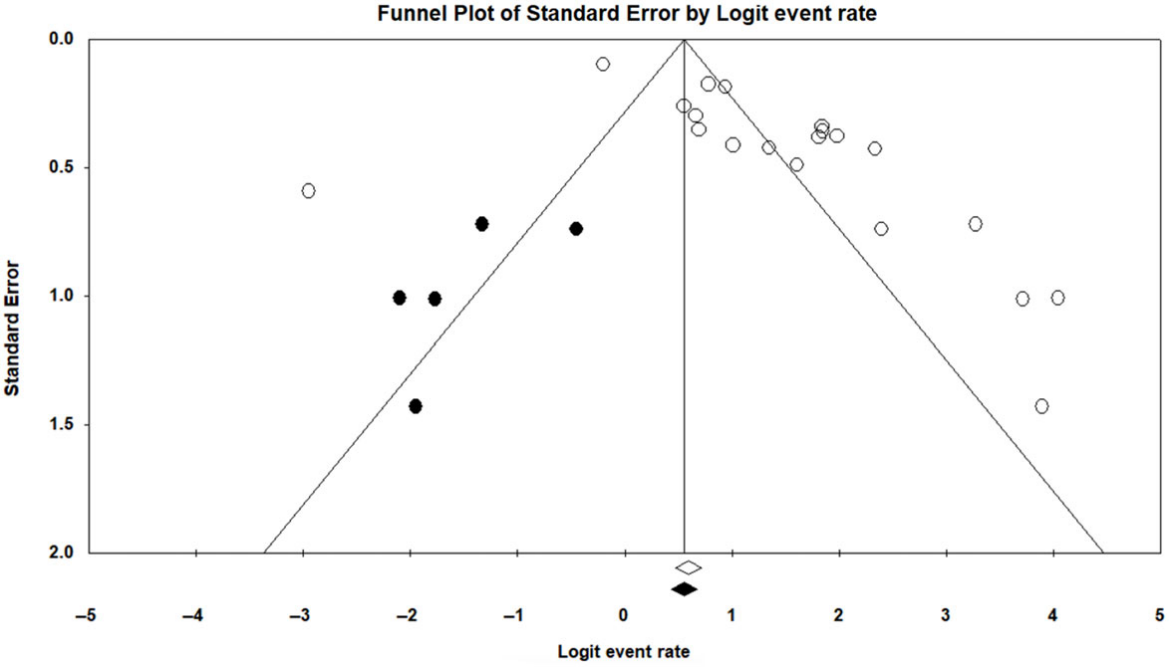
Funnel plot of treatment completion with imputed studies. Note: Imputed studies are shown in black.

Visual assessment of the funnel plot for treatment completion suggests moderate publication bias (see Fig. 3 for the funnel plot with unpublished studies imputed). Quantitative assessments using Begg’s rank correlation analysis trended toward significance (p = 0.08), while assessment of Egger’s test of the intercept was statistically significant (p < 0.01), suggesting some publication bias. We used the trim-and-fill method to determine the number of missing studies that would be needed to correct the asymmetry (see Fig. 3); this method suggests 5 imputed studies; however, current literature indicates that substantial heterogeneity in effect sizes (as is present in this analysis) seriously impairs the power of the trim-and-fill method, since the plot’s asymmetry may be confounded by heterogeneity [83–85]. Therefore, these results should be interpreted with caution. Although established statistical methods for assessing publication bias in meta-analysis are traditionally used to examine bias in treatment effects, these results may also have implications for examining bias in study completion rates. In particular, studies with low completion rates may be likely to have fewer positive treatment effects, increasing the probability that results will not be published.

## Discussion

This systematic review and meta-analysis represent the first attempt to quantitatively review and synthesize behavioral health treatment completion data among JIY enrolled in treatment studies. This meta-analysis aimed to derive a more accurate estimate of treatment completion among JIY and better understand issues related to treatment engagement strategies and barriers to treatment completion. Findings indicated relatively high rates of treatment completion, with 82.6% of youth who initiated treatment completing treatment according to the treatment’s specified completion criteria. Our findings suggest when some systemic barriers to treatment (i.e., low treatment availability, difficulty identifying appropriate treatment, treatment cost) are removed by the presence of providing treatment within a study, JIY may not be any more difficult to engage in treatment than the general adolescent population.

One aim of this investigation was to begin to disentangle the influence of barriers to behavioral health treatment from the challenge of helping JIY complete treatment. That is, when behavioral health treatment is available and accessible (i.e., where treatment is provided through the study and JIY have been determined to be in-need of treatment), is it still difficult for JIY to complete treatment? This does not seem to be true in these studies, as the majority did complete treatment. Another aim of this investigation was to identify moderators of treatment completion. Type of treatment (i.e., family, group, individual) was a significant moderator, with treatments that provided family- or group-based treatment having higher rates of treatment completion compared to treatment groups that provided individual treatment. This is consistent with prior research findings that family-based treatments are associated with greater engagement in care [17,86]. Some have argued that the greater engagement associated with family and group-based interventions for adolescents may relate to the social components inherent to these types of interventions [87]. Adolescence, marked by many important socio-developmental milestones, typically includes dynamic changes to the role and identification of supportive others in their lives [16,88,89], and this may be particularly true for JIY [90]. For adolescents who may not have a strong, existing support network, group- and family-based interventions may be one desirable method of facilitating or improving these social connections, making them more engaging and desirable. These types of interventions may align more with helping adolescents meet important developmental milestones than individual modalities might. Even further, qualitative literature examining perspectives from youth, caregivers, treatment providers, and juvenile justice personnel consistently suggests that caregiver involvement is essential to achieve youth uptake in treatment and maintain engagement [91,92]. Thus, family-focused treatments may increase rates of treatment completion by increasing caregiver involvement and support.

### Influence of Publication Bias and Study Quality

Analyses indicated some likelihood that publication bias has influenced the results; funnel plots show imputed studies with lower effect sizes than those reported by published studies, which suggests rates of treatment completion may be lower. While funnel plots should be interpreted with caution given the high level of heterogeneity present in the data [93,94], 6 studies that otherwise met eligibility criteria for inclusion in these analyses did not report full data on treatment completion and therefore could not be included in these analyses. Further, the majority of studies did not sufficiently describe withdrawals and dropouts, making it difficult to identify reasons or demographic characteristics predictive of treatment noncompletion.

### Moderators not Supported by the Current Investigation

Hypotheses regarding the intervention focus (i.e., substance use disorders, conduct disorders, etc.) and racial or ethnic minority participants were not supported as moderators. In the case of intervention focus, the majority of studies (*n* = 15, 71.4%) were focused on addressing substance use disorders or problematic substance use, so it is possible that our analyses were underpowered to detect differences between studies with different intervention foci. It is less likely that moderator analyses of the prevalence of racial or ethnic minority participants were underpowered, given that there was a wide range of study participants who identified as racial or ethnic minorities. Previous research suggests that minority youth face significant barriers to *accessing* behavioral health treatment but fewer barriers when that treatment is available and accessible [20]. For example, a systematic review of literature on referrals to behavioral health services from the juvenile justice system [95] finds that a majority of the 26 articles reviewed reveal at least some evidence of racial disparities in decisions to refer youth. Thus, disparities in “utilization” may be more appropriately named disparities in access.

The inclusion of interventions to increase treatment engagement was not a significant moderator of treatment completion. Given the broad heterogeneity in treatment engagement interventions provided by studies included in this analysis, it may be important to conduct additional research assessing the success of such interventions.

Another potentially important moderator that should be examined in future studies is mandated treatment (i.e., when youth are required by the court to seek outpatient behavioral health treatment). We did not examine this as a moderator in the current study since only 6 studies reported information about whether or not youth were court-mandated to engage in behavioral health treatment. Of the 6 that reported on treatment mandates, results were highly heterogeneous (i.e., 42–100%). Literature on the effectiveness of court mandates for outpatient treatment is mixed and may depend in particular on the variability of court mandates by jurisdiction [24,96]. However, recent research [97] found that youth who attended treatment at court-direction (compared to voluntarily) demonstrated higher rates of SUD treatment completion; this variable should be further examined as a possible moderator in future research. One limitation of this analysis is that, due to the small number of control groups providing services as usual that reported data on treatment completion (k = 2), we were unable to consider experimental treatment group as a moderator of treatment completion. It is important for studies examining treatment completion in JIY to report this data for all treatment groups. Future systematic reviews should consider this factor in their analyses.

### Implications and Recommendations for the Future

Overall, our findings suggest that existing high-quality studies of behavioral health treatment among JIY have generally achieved high rates of treatment completion. While included studies were not limited by presenting problem, all the studies included in this meta-analysis examine treatment of either substance use or problematic sexual behavior. Notably, the majority of treatments provided in these studies (Multisystemic Therapy, MET-CBT, Multidimensional Family Therapy) are not specific to inappropriate sexual behavior or substance use and are frequently provided to youth and families with a broad range of diagnoses. Thus, we expect that these results are generalizable to many JIY and families receiving behavioral healthcare. However, it is likely helpful for the field to consider what the effectiveness of other evidence-based practices (e.g., behavioral activation) may look like for JIY. Expanding access to evidence-based treatment and helping youth and their families remain engaged in services will both be critical challenges for researchers, policymakers, juvenile justice professionals, and community mental health administrators. Based on the results of our systematic review and meta-analysis, we make three recommendations for future research, implementation, and practice of behavioral health interventions for JIY:

1. Researchers should place a greater focus on measuring and reporting treatment completion. This includes thoroughly describing dropouts and withdrawals and reporting treatment completion or “dose” criteria, to ensure that estimates of treatment efficacy are not confounded by youth engaging in different amounts of treatment.

2. Given the potentially important role that treatment mandates play in the referral of JIY to behavioral health treatment, researchers should attempt to document the nature of youth’s justice involvement and whether they have been mandated to participate in outpatient treatment (even if study participation is not mandatory).

3. Researchers should consider utilizing interventions that include family- or group-based services to improve rates of treatment completion.

## Supplementary material

For supplementary material accompanying this paper visit https://doi.org/10.1017/cts.2022.418. click here to view supplementary material
