# Supplementary material for: Treatment completion among justice-involved youth engaged in behavioral health treatment studies in the United States: A systematic review and meta-analysis
Source: J Clin Transl Sci. 2022 Jun 13;6(1):e86. doi: 10.1017/cts.2022.418 (PMC9389282; doi:10.1017/cts.2022.418)
Supplement: Supplementary file 1 [file S2059866122004186sup001.docx]

Supplementary Table. **Primary Study Quality**

|  | Study Quality Variables | | | | | | | |
| --- | --- | --- | --- | --- | --- | --- | --- | --- |
|  | Differences between refusers and non-refusers at baseline | Reasons for non-participation | Utilizes intent-to-treat analyses | Reports treatment completion criteria | Reports N who completed treatment | Detailed description of those who did not complete | Demographic differences in attrition & treatment completion by group | Differences in attrition after random assignment |
| Borduin et al., 2009 | No | No | Yes | Yes | Yes | No | No | Yes |
| Burrow-Sanchez et al., 2015 | No | Yes | Yes | Yes | Yes | No | No | Yes |
| Dakof et al., 2015 | No | No | Yes | Yes | Yes | No | No | Yes |
| Henderson et al., 2016 | No | No | Yes | Yes | Yes | No | Yes | Yes |
| Henggeler et al., 1999 | Yes | No | Yes | Yes | Yes | No | Yes | Yes |
| Henggeler et al., 2015 | No | No | Yes | Yes | Yes | No | No | Yes |
| Kaminer et al., 2019 | No | No | Yes | Yes | Yes | Yes | NA | NA |
| Letourneau et al., 2009 | No | No | Yes | Yes | Yes | No | No | Yes |
| Schaeffer et al., 2014 | No | No | Yes | Yes | Yes | No | No | Yes |
| Sharkey et al., 2010 | No | No | No | Yes | Yes | Yes | NA | NA |
| Silovsky et al., 2019 | No | Yes | Yes | Yes | Yes | No | No | NA |
| Tolou-Shams et al., 2017 | No | No | Yes | Yes | Yes | Yes | Yes | Yes |
| Walker et al., 2019 | No | No | No | Yes | Yes | No | No | NA |
